# Virological and Histopathological Findings in Boars Naturally Infected With Porcine Reproductive and Respiratory Syndrome Virus Type 1

**DOI:** 10.3389/fmicb.2022.874498

**Published:** 2022-05-11

**Authors:** Kasper Pedersen, Sophie Amalie Blirup-Plum, Charlotte Sonne Kristensen, Lise Kirstine Kvisgaard, Lotte Skade, Henrik Elvang Jensen, Lars Erik Larsen

**Affiliations:** ^1^SEGES Danish Pig Research Centre, Aarhus, Denmark; ^2^Department of Veterinary and Animal Sciences, University of Copenhagen, Frederiksberg, Denmark

**Keywords:** boar, immunohistochemistry, natural infection, porcine reproductive and respiratory syndrome virus, RT-PCR

## Abstract

Major geographical transmission of porcine reproductive and respiratory syndrome virus (PRRSV) occurs *via* semen when a boar stud is infected. This happened in Denmark in 2019, providing an opportunity to compare previous experimental PRRSV boar studies with natural PRRSV-1 infection in boars. The aim of this study was to investigate the association between the presence of PRRSV RNA in serum, semen, testicles, and epididymis of boars naturally infected with PRRSV and to describe the histological lesions in the testes and epididymis combined with direct visualisation of PRRSV-infected cells by immunohistochemical staining (IHC). The exact timing of infection of each boar was not determined, but based on serology the boars were divided into two groups: acute and late infections. All boars included were sampled the same day. In this study, 35 boars and 10 healthy boars from another PRRSV-negative boar stud were included as histological controls. PRRSV RNA was found most often in serum (51%) and least frequently in semen (22%) and was more often detected in the reproductive tract in the acute phase of infection (*p* < 0.0001; RR: 2.58). Mononuclear cells and multinuclear giant cells were present in the adluminal compartment of the testis and epididymis in PRRSV-infected boars, but not in control boars (*p* < 0.05), which supports the hypothesis that macrophages are involved in the venereal spread of the virus.

## Introduction

Porcine reproductive and respiratory syndrome viruses (PRRSVs) are highly contagious viruses affecting pigs globally. The pronounced genetic differences among strains have resulted in the current classification of the virus within the new genus *Betaarterivirus* (family *Arteriviridae*, order *Nidovirales*) as two distinct viral species, *Betaarterivirus suid 1* (for PRRSV-1; subgenus *Eurpobartevirus*) and *Betaarterivirus suid 2* (for PRRSV-2; subgenus *Ampobartevirus*) (Brinton et al., 2018). Infected pigs excrete PRRSV through respiratory aerosols, saliva, urine, faeces, and semen (Christopher-Hennings et al., 1995b,^2001^; Wills et al., 2003; Plut et al., 2020). Venereal transmission of PRRSV by artificial insemination has been documented in experimental studies (Swenson et al., 1994; Gradil et al., 1996; Nielsen et al., 1997; Prieto et al., 1997; Guérin and Pozzi, 2005) and in the field (Yaeger, 1993; Nathues et al., 2016). Infection of boars at boar studs used for collection of semen for artificial insemination is of particular concern because the virus can be widely disseminated to a large number of sow herds, often with devastating effects (Nathues et al., 2016). In July 2019, a PRRSV-free boar stud in Denmark was infected with a recombinant strain of PRRSV (Kvisgaard et al., 2020). Based on the results of the routine surveillance samplings performed at 14-day interval, combined with diagnostic samples taken after the outbreak was discovered, it was estimated that the virus had circulated among the boars for approximately 3 weeks before PRRSV specific antibodies were detected and the distribution of semen was terminated. In fact, a follow-up study revealed that at least 36 sow herds became infected by PRRSV-1 contaminated semen from this boar stud during this period (Kristensen et al., 2020; Kvisgaard et al., 2020).

It is still unclear how the PRRS virus enters the semen, but it has been suggested that the pathogenesis of PRRSV from initial infection to contaminated semen goes through systemic hematogenously distributed macrophages that relocate to the interstitium of the testes (Sur et al., 1997). PRRSV-2 causes desquamation of seminiferous epithelial cells (Sur et al., 1997), possibly by apoptosis of bystander cells (Sirinarumitr et al., 1998). Thus, PRRSV-infected macrophages may relocate to the seminiferous tubules or release PRRSV directly into the seminiferous tubules (Sur et al., 1997; Christopher-Hennings et al., 1998; Prieto and Castro, 2005).

The pathological impact and dynamics of PRRSV infection in boars have been studied only in a few experimental studies (Prieto and Castro, 2005). Yet, to the best of our knowledge, no studies have examined the impact of PRRSV on boars during acute natural infections in the field. It was decided to slaughter all boars at the boar stud, and this provided a unique possibility to obtain material from infected boars at different stages of infection. This study describes the results of examinations of viral distribution and histopathological changes in the reproductive tissue of a subset of the infected boars and relates these findings to the presence of PRRSV-1 in semen, tissue, and serum from the same boars.

## Materials and Methods

### Animals, Grouping, and Time Frame

Although the exact time of infection for each boar has not been defined, it was estimated that PRRSV-1 was introduced into the boar stud between 1 July and 10 July 2019 (Kvisgaard et al., 2020). PRRSV was initially detected in samples taken on 10 July by test of the routine surveillance samples by real-time reverse transcription polymerase chain reaction (RT-PCR). Thus, 10 July was defined as day 0 (d0) in this study, and the last boars were sampled on 8 August, corresponding to day 29 (d29) in the study (study timeline is illustrated in Supplementary Figure 1). Boars included in this study were selected based on subjective estimations of time of infection based on the level (S/P values) of PRRSV specific antibodies and PRRSV detections in each section of the boar stud.

**FIGURE 1:**
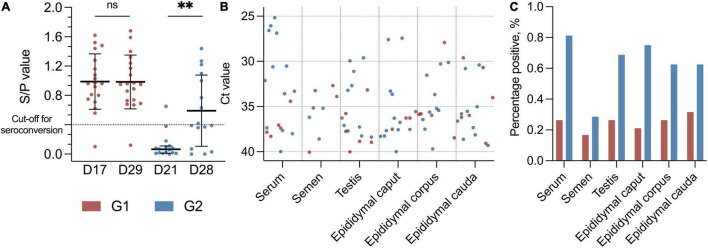
**(A)** Scatter dot plot with distribution of boars with specific PRRSV antibodies with S/P value on the *Y*-axis. Black lines indicate mean with SD (D17, D29, and D28) and median with 95% CI (D21), and asterisks (**p < 0.01) and “ns” indicate *p*-value summary. Cut-off for seroconversion is indicated with a horizontal dotted line. Early seroconversion as G1 (red dots) and late seroconversion as G2 (blue dots). G1 at day 17 (D17) and day 29 (D29) (*p* = 0.46). G2 at day 21 (D21) and day 28 (D28) (*p* < 0.01, Mdn: 0.40, 95% CI: 0.26–0.69). **(B)** Scatter dot plot with Ct values (*Y*-axis) from the PRRSV-1 RT-PCR with different tissues (*X*-axis) from the PRRSV-1-infected boars. Red dots indicate early PRRSV-infected boars (G1), and blue dots indicate later PRRSV-1-infected boars (G2), and sample material is from day 29 and day 28 in the study, respectively. In RT-PCR, 117 data points as negative values are not included. **(C)** Bar plot with percentage positive results in the PRRSV-1 RT-PCR (*Y*-axis) from different tissues (*X*-axis). Colours indicate group, with red as G1 and blue as G2.

Of the PRRSV-infected boars, four Landrace, three Yorkshire, and 28 Duroc boars were included in the study. The boars were between 248 and 737 days old, with a mean age of 468 days (15.4 months). The boars were allocated into two groups, designated as early (G1) and late (G2), based on day of seroconversion (Figure 1A). This was coordinated with the sectional division at the boar stud, since all boars in G1 were housed in section two and all boars in G2 were housed in sections four and five (Supplementary Figure 2). For the histopathology, testis and epididymis from ten healthy boars (designated control boars) from another boar stud were included.

### Sample Collection

Serum from all boars was collected at two time points. In the early seroconversion group (G1), serum was collected during routine surveillance on day 17, and serum from boars in the late seroconversion group (G2) was collected on day 21. At study termination, serum from all boars in G1 and G2 was also collected at the abattoir immediately after CO_2_-stunning during bleeding on days 29 and 28, respectively. Semen was collected the day before the boars were sent to the abattoir, corresponding to day 28 for G1 and day 27 for G2. Reproductive tissue (testis with epididymis) was collected into separate bags on the slaughter line as the first touch after scalding. The tissue was transported directly within 2 h to the SEGES Swine Diagnostic Laboratory, where professional pathologists collected pieces of the testis, caput, corpus, and cauda epididymis. Tissue samples for histological examination were fixated in 10% neutral formalin buffer, and samples for the RT-PCR were stored at -80°C until further diagnostics.

### Laboratory Analysis

Initially, serum was separated by centrifugation of the blood samples at 2,600 × *g* for 10 min. Diluted (1:40) serum samples were tested for antibodies against PRRSV with a commercially available enzyme-linked immunosorbent assay (ELISA) (IDEXX PRRS X3 Ab Test, Idexx Hoofddorp, Netherlands) using the instructions recommended by the manufacturer. Results were expressed as a sample to positive control (S/P) ratio, and the cut-off was set at S/P ≥ 0.4. Total RNA in serum and semen was extracted using the QIAcube HT robot (QIAGEN GmbH, 70724 Hilden, Germany) followed by a purification step of 200 μl aliquots utilising the protocol “Cador Pathogen 96 QIACube HT V3.” Tissue samples were extracted using the QIAcube^®^ Connect robot (QIAGEN GmbH, 70724 Hilden, Germany). In short, 70 mg of each tissue sample was transferred to an Eppendorf tube (Sigma-Aldrich, Eppendorf Safe-Lock microcentrifuge tubes, 2.0 ml) with 1,400 μl RTL buffer including 1% β-mercaptoethanol and a stainless-steel bead (QIAGEN, stainless steel beads, 5 mm) and processed in a TissueLyser II (Qiagen, TissueLyser II) for 3 min at 30 kHz. The suspension was centrifuged for 3 min at 9,700 × *g* (Espresso Personal Centrifuge, Thermo Fisher). Of note, 600 μl supernatant was transferred to a Sarstedt tube (Sarstedt 2.0 ml SC Micro Tube PCR-PT), and RNA was extracted using RNeasy^®^ Mini Kit (QIAGEN GmbH, 70724 Hilden, Germany) with an elution volume of 60 μl. In extractions, known PRRSV-positive and PRRSV-negative controls were included. The full procedure was performed as previously described (Kvisgaard et al., 2021). All serum, semen, and tissue samples were analysed using RT-PCR as previously described (Wernike et al., 2012). Samples were considered positive for PRRSV-1 if the cycle threshold (Ct) value was ≤ 40.0.

### Sequencing

To confirm the virus strain, testicular tissue samples were selected for Sanger sequencing of partial ORF2 (nucleotide no. 117–684) and full length ORF5. The sequencing procedure has previously been described (Kvisgaard, 2013; Kvisgaard et al., 2020).

### *In vitro* Propagation of Virus

A previously used protocol for propagation of virus in cell culture was used (Kvisgaard et al., 2020). In short, a 10% homogenate of testicular tissue was prepared in Minimum Essential Medium (MEM, Gibco) with 10% Penicillin-Streptomycin (Sigma), homogenised as previously described, centrifuged for 2 min at 12,000 × *g* (Espresso Personal Centrifuge, Thermo Fisher), and the supernatant was passed through a 0.45-μm filter (Millex). Semen samples were diluted 1:1 in MEM with 10% Penicillin-Streptomycin and filtered through a 0.45-μm filter (Millex). When 90–95% confluency of the MARC 145 (9th passage) cells was observed in the T25 flasks, the cells were inoculated with the sample material for 2 h. The inoculum was replaced with cell media of MEM, 5% foetal bovine serum (Sigma), 1% Penicillin-Streptomycin and Glutamine, and 1% MEM Non-Essential Amino Acids (Gibco) and incubated for 5 days in a humanised atmosphere (37°C and 5% CO_2_). The cells were monitored daily for cytopathic effect (CPE). Virus isolates were harvested after one freeze/thaw cycle, and cell debris was removed by centrifugation at 500 × *g*, 10 min RT. Two further passages were performed with 200 μl 1st passage isolates and 3 ml cell media. RNA was extracted from cell isolates in each passage as previously described and assessed for virus propagation with PRRSV RT-PCR.

### Histopathology

One tissue section from the right testis and the right epididymis (caput, corpus, and cauda) of each of the 35 PRRSV-1-infected boars and 10 controls was cut into representative pieces and embedded in paraffin. Tissue sections of 4–5 μm for the histological examination were stained with haematoxylin and eosin (H&E).

### Immunohistochemistry

Tissues from PRRSV-infected boars and 10 control boars were immunohistochemically (IHC) stained for PRRSV-1. For immunostaining, the tissue sections were mounted on adhesive glass slides (Thermo Scientific, Menzel Gläser, United States). A rabbit anti-PRRSV-1 nuclear protein antiserum, PRSNP11-S, (Alpha Diagnostic Intl. Inc.) was used. The immunostaining technique was performed using the Ultravision LP Detection system HRP (Thermo Scientific, Cheshire, United Kingdom). After dewaxing the sections, antigen retrieval was carried out by treatment with a proteinase K solution (Sigma P6556, 5.0 mg/ml) for 30 min at 45^°^C. This was followed by blocking of endogenous peroxidase activity by 3% H_2_O_2_ for 10 min and blocking of unspecific binding by Ultra V Block for 5 min (AH diagnostics, TL-125-HL). The tissue sections were then incubated with the primary antibody PRSNP11-S [diluted 1:800 in 1% bovine serum albumin/tris-buffered saline (TBS)] for approximately 20 h at 4^°^C. The primary antibody enhancer was added for 20 min, the horseradish peroxidase (HPR) polymer for 30 min, and diaminobenzidine (DAB) (Cell Marque 957D-40) for 10 min. Throughout the protocol, with the exception of the step between Ultra V blocking of unspecific binding and the application of the primary antibody, slides were washed in TBS, pH 7.6. Counterstaining was performed in Mayer’s haematoxylin (VWR international), and the sections were rinsed in distilled water. Coverslips were mounted with glycerol gelatine. Positive and negative controls were run on PRRSV-1-positive lung tissue (Supplementary Figure 9A). Negative controls included deletion of the primary antibody and substitution of the primary antibody with a nonsense antibody (DAKO X0903).

### Statistical Analysis

Data management was performed with Microsoft^®^ Excel Version 16.49, and the statistical analysis was performed in Graph Pad Prism 9 Version 9.1.2. Results from the ELISA are both processed as quantitative and dichotomous (positive/negative) according to the cut-off value, and RT-PCR results are considered dichotomous (positive/negative) according to the cut-off value. First, all data were analysed using a descriptive statistical method and presented with mean or median, depending on Gaussian distribution and difference in variances. In the statistical analysis, the level of statistical significance (*p*) was 5%. A one-tailed paired Student’s *t*-test and a Wilcoxon matched-pairs signed rank test were used to compare S/P values at day 17 with day 29 in G1, and day 21 with day 28 in G2, respectively. A two-sided Fisher’s exact test was used in the binary comparison of seroconverted and viraemic boars as well as PRRSV-1 RNA in semen and viraemic boars. Furthermore, this test was used to compare the presence of PRRSV-1 RNA in serum, semen, and testis, and in different anatomical locations in the epididymis in each group (G1/G2) and the presence of intraluminal cells (mononuclear cells/macrophages) in case vs. control. A two-sided Fisher’s exact test was also used in the comparison of PRRSV seroconverted and PRRSV-1 semen positive boars. Two-sided chi-square test was used for the comparison of PRRSV-1 RNA total positive samples in G1 and G2, testis vs. epididymis (caput, corpus, and cauda) and the presence of PRRSV-1 RNA in epididymis in total (caput, corpus, and cauda) as well as the detection in caput, corpus, and cauda epididymis.

## Results

None of the 35 naturally PRRSV-infected boars included in this study developed clinical symptoms of PRRSV at any time during the study.

### Serology

As a group, the boars in G2 had a significant increase in antibody level between days 21 and 28 (*p* < 0.01, *Mdn*: 0.40, 95% CI 0.26–0.69) (Figure 1A). No significant changes in antibody levels between days 17 and 29 were detected for G1 (*p* = 0.46, *M*: 0.00 SD 0.18). At the termination of the study (d29), 18 of 19 (95%) boars in G1 were positive for specific antibodies toward PRRSV-1, and the group’s mean S/P value was 0.99 (SD: 0.38) compared to 0.98 (SD: 0.37) at day 17 (Figure 1A). The boar that did not seroconvert throughout the study was PRRSV-1-positive in serum by RT-PCR at day 0. All but one of the boars in G2 were seronegative at day 21 (*Mdn*: 0.07, SD 0.17), and 8 of 16 (50%) of these boars had seroconverted at the termination of the study (d28) (*M*: 0.59, SD 0.49) (Figure 1A). Four of the G2 boars had an S/P value that was just below the cut-off value (S/P: 0.36–0.39) at day 28.

### PRRSV-1 Detection by Real-Time Reverse Transcription Polymerase Chain Reaction

In total, 207 samples, including serum, testicle, and epididymis (caput, corpus, and cauda), were collected at slaughter on days 28 and 29. In addition, semen samples were collected from 32 of the 35 boars at the day before slaughter. Three of the boars were not able to deliver semen. One of them was from the group with early seroconversion (G1), and two of them were from the group with late seroconversion (G2). All samples were tested for PRRS virus by RT-PCR. Overall, 88 of 207 (43%) of the samples were positive for PRRSV-1 independently of material investigated (Figures 1B,C and Supplementary Table 1).

**FIGURE 2:**
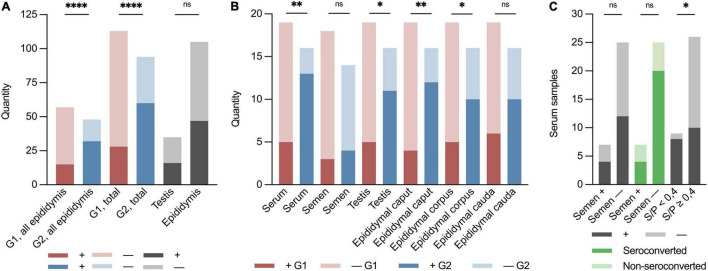
Binary (±) stacked bar plots of sample material from the PRRSV-1-infected boars at the boar stud in Denmark 2019. “ + ” indicates the presence of PRRSV-1 RNA, and “–” indicates not detected by RT-PCR. Red indicates early infected boars, G1; blue indicates later infected boars, G2; and dark grey indicates positive samples in total of testis, epididymis, semen, and serum. Light grey indicates negative samples. Dark green indicates seroconverted boars and light green indicates non-seroconverted boars. **(A)** Groupwise division of all epididymal samples in G1 and G2 (*p* < 0.0001; RR 2.53; 95% CI 1.61–4.15), early (G1) and late (G2) seroconversion of results in RT-PCR of all sample material (*p* < 0.0001; RR 2.58; 95% CI 1.83–3.70), and of all testis and epididymis samples (*p* = 0.92). **(B)** Groupwise division of PRRSV-1-viraemic and negative boars at slaughter (*p* < 0.01; RR 3.09; CI 1.53–7.06), PRRSV-1-infected and negative semen (*p* = 0.67), PRRSV-1-positive and negative testis (*p* < 0.05; RR 2.61; 95% CI 1.22–6.11), epididymal caput (*p* < 0.01; RR 3.56; CI 1.57–9.10), corpus (*p* < 0.05; RR 2.38; CI 1.08–5.64), and cauda (*p* = 0.09). **(C)** Quantity of serum samples on the *Y*-axis. *X*-axis shows semen PRRSV-1-positive (+) or PRRSV-1-negative (–) (viraemic: *p* > 0.99 and seroconverted: *p* = 0.22), non-seroconverted (S/P < 0.4), and seroconverted boars (S/P ≥ 0.4) (*p* < 0.05; RR 2.30; 95% CI 1.28–4.07). Asterisks and “ns” indicate *p*-value summary (*p < 0.05, ^**^p < 0.01, and ^*⁣*⁣**^p < 0.0001).

One-fourth (1/4) of the samples from the G1 boars and approximately 2/3 of the samples from the G2 boars were positive for PRRSV-1 virus at slaughter. Thus, significantly more boars in G2 were RT-PCR PRRSV-positive than the boars in G1 (*p* < 0.0001, RR 2.58, 95% CI 1.83–3.70) (Figure 2A). Test of serum revealed that 18 of 35 (51%) of the boars were viraemic at slaughter. In total, 13 of 16 (82%) boars in G2 were viraemic at slaughter compared to 5 of 19 (26%) in G1 at slaughter (*p* < 0.01; RR 3.09 CI 1.53–7.06) (Figures 1B,C, 2A). Conversely, eight of nine boars that tested negative for antibodies were viraemic at slaughter (*p* < 0.05; RR 2.30 95% CI 1.28–4.07) (Figure 2C). Semen from 7 of 35 boars (22%) tested positive for PRRSV-1 (Figures 1B,C). Three of the PRRSV-1-positive semen samples were from boars in G1 and tested negative in serum at slaughter. The remaining semen-positive boars were from G2 and were all viraemic, but no significant difference was detected between G1 and G2 (*p* = 0.67*)* (Figure 2B). Of the seven boars with PRRSV-1-positive semen, four were viraemic (*p* > 0.99), and three of these were seronegative (*p* = 0.22) (Figure 2C). In testis samples, 16 of 35 (46%) were PRRSV-1-positive (Figures 1B,C), while 47 of the 105 (45%) samples from the epididymis were positive for PRRSV-1 (Figures 1B,C, 2A) (*p* = 0.92). In the testis of the G1 boars, 5 of 19 (26%) were positive vs. 11 of 16 (69%) of the boars in G2 (Figure 2B). Thus, more G2 boars tested positive in the testis compared to G1 boars (*p* < 0.05, RR 2.61, 95% CI 1.22–6.11). In G1, 4 of 19 (21%), 5 of 19 (26%), and 6 of 19 (32%) tested positive in the caput, corpus, and cauda epididymis, respectively, while 12 of 16 (75%), 10 of 16 (63%), and 10 of 16 (63%) were positive in G2 (caput: *p* < 0.01; RR 3.56; CI 1.57–9.10, corpus: *p* < 0.05; RR 2.38; CI 1.08–5.64, cauda: *p* = 0.09) (Figures 1B,C, 2B). In samples of epididymis, 15 of 57 (26%) of the boars in G1 and 32 of 48 (67%) of the boars in G2 were positive for PRRSV-1 (Figure 2A). Similar to the testis test, significantly more G2 boars were PRRSV-positive in the epididymis compared to the G1 boars (*p* < 0.0001; RR 2.53; 95% CI 1.61–4.15). Comparisons between the tested parts of the epididymis for both G1 and G2 revealed that 16 of 35 (46%), 15 of 35 (43%), and 16 of 35 (46%) of the epididymal caput, corpus, and cauda, respectively, tested positive for PRRSV-1. No significant differences were observed between the three sections (*p* = 0.96).

### Confirmation of Viral Strain by Sanger Sequencing

To confirm the virus strain circulating in the boars, partial ORF2 and ORF5 Sanger sequencing was carried out. These two fragments are located on each side of the recombinant breakpoint in the recombinant virus strain previously found in boars from the boar stud (Kvisgaard et al., 2020). Pairwise nucleotide comparisons of sequenced fragments showed that the virus was in fact the new recombinant strain with 99.60–100.00% identity in partial ORF2 and 99.83–100.00% identity in ORF5 to the previous isolated recombinant virus strain (MN603982).

### *In vitro* Propagation of Virus

To clarify if PRRSV RT-PCR positive semen samples and matched testicular tissue contained infectious viruses, propagation of the virus in cell culture was attempted for five of six PRRSV RT-PCR positive semen samples and for one PRRSV RT-PCR negative semen sample that tested PRRSV RT-PCR positive in the testicular tissue. No CPE was observed during the three passages for any of the samples. Propagation of PRRSV did not succeed with selected samples because of declining viral RNA detection in RT-PCR during passages (Supplementary Table 2), and no samples were positive in the third passage.

### Histopathological Changes in the Testis and Epididymis

#### Control Boars

All but one of the testis sections presented with dilated lymphatic vessels and interstitial oedema. In all epididymal sections, interstitial oedema was present (Supplementary Figure 3A). Mononuclear cell infiltration (perivascular and periductular) as well as hyperaemia were observed in half of the sections. Another frequent (7/10) finding was pyknosis and karyorrhexis of the spermatocytes in a few seminiferous tubules. Two boars had focal tubular degeneration, and one boar had general tubular atrophy. Mononuclear cell infiltration (perivascular and periductular) (Supplementary Figure 3C), interstitial hyperaemia, and smooth muscle hypertrophy of the epididymal ducts were observed sporadically in the ten animals. Vacuolisation of the epithelial cells was seen in three boars (Supplementary Figure 3B). All tissue samples from the ten control boars were PRRSV-negative in IHC (Supplementary Figure 9B).

#### PRRSV-1-Infected Boars

All boars presented with interstitial oedema in both testis (Supplementary Figure 4A) and epididymis (Supplementary Figure 4B), and dilated lymph vessels in the testis (Supplementary Figure 4C). In 18 of the testis sections, pyknosis and karyorrhexis of the spermatocytes in a few seminiferous tubules were present (Supplementary Figure 4D), and five of these had areas with absent nuclei of the Leydig cells (Supplementary Figure 4E). Mononuclear cell infiltration (perivascular and periductular) was observed in 23 and 19 (65.71 and 54.29%) of the testis (Supplementary Figures 5A,B) and epididymis (Supplementary Figures 5C,D) samples, respectively. Interstitial hyperaemia was present in 16 and 11 of the 35 (45.71 and 31.43%) testis (Supplementary Figure 4F) and epididymis sections, respectively, and focal tubular degeneration was present in five (14.29%) testis sections (Supplementary Figures 6A,B). In 18 (51.43%) boars, there was interstitial smooth muscle hypertrophy of the epididymal ducts Supplementary Figures 6C,D, and nine (25.71%) boars had severe vacuolisation of the epithelial cells in the epididymis (Supplementary Figure 7A).

**FIGURE 3:**
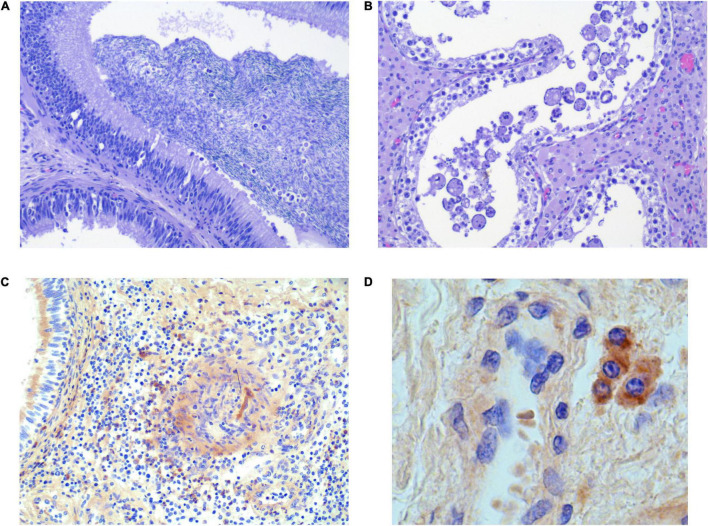
Selected histological sections of testis **(B,D)** and epididymis **(A,C)** from PRRSV-1-infected boars, stained with H&E **(A,C)** and IHC **(C,D)**. **(A)** Intraluminal mononuclear cells [infiltrating the epithelium (arrows)]. **(B)** Intraluminal swollen/enlarged/giant cells. **(C)** Few focally distributed infected macrophages in the testicular interstitium. **(D)** Few focally distributed infected macrophages in the interstitium of epididymis (40×).

An essential finding was the presence of intraluminal mononuclear cells in five epididymal sections from each of the groups (Figure 3A). Giant cell formation was seen in four sections from G1, two of which were collected from the boars that also had intraluminal mononuclear cells in the epididymis. One of these boars from G1 with intraluminal cells in the epididymis (Supplementary Figure 7B) also had intraductal MGC formation in a testis section (Figure 3B). Three and one of the 35 samples from the epididymis and testis, respectively, presented with a few focally distributed PRRSV-1-infected macrophages in the interstitium, discovered by IHC (Figures 3C,D and Supplementary Figure 8). Overall, mononuclear cells and/or MGC were more often detected in the reproductive tissue of PRRSV-1-infected boars compared to reproductive tissue from control boars (*p* < 0.05, CI 95% 1.15–∞).

## Discussion

This study is an extension of previous studies performed in relation to the PRRSV-1 infection at a boar stud in Denmark in 2019 described by Kristensen et al. (2020) and Kvisgaard et al. (2020, 2021). PRRSV-1 infection of the boars was confirmed by (i) detectable specific antibodies against PRRSV, (ii) detection of PRRSV-1 RNA in sample material, and (iii) discovery of PRRSV-1 positive cells by IHC. The primary objective of this study was to investigate potential histological lesions in the testes and epididymis of boars naturally infected with PRRSV-1, together with direct detection of PRRSV-1-infected cells. The second objective was to investigate the distribution of PRRSV-1 in the testis and epididymis and to analyse the association between the presence of PRRSV-1 RNA in serum, semen, testicles, and epididymis. This information is important for the design of surveillance programs in boar studs.

None of the boars showed clear signs of disease following infection, which is in accordance with the results of experimental infections of adult non-pregnant animals (Prieto and Castro, 2005) and other reported outbreaks in boar stations (Nathues et al., 2016).

The results of this study are in accordance with experimental and epidemiological data that provide evidence for the venereal transfer of PRRSV by semen and confirm previous findings that natural PRRSV infection of adult boars may also result in the transfer of PRRSV *via* semen (Nathues et al., 2016). PRRSV-1 RNA was most frequently detected in serum (51%), and least frequently in semen (22%), which corresponds to the results of a previous study where 20% of the experimentally infected boars (*n* = 20) shed PRRSV-1 in semen (Prieto et al., 2003).

At slaughter, eight seropositive boars tested negative in all tested samples (seven from the early infected group, G1, and one from the later infected group, G2). It seems conceivable that PRRSV-1 RNA is more likely to be present in the reproductive tract in the acute phase of infection (*P* < 0.0001). Interestingly, three boars were still shedding virus in semen despite the fact that they had seroconverted and were non-viraemic. This has a significant impact on the sensitivity of surveillance programs, which often rely on serum tests for declaration of negative animals. In summary, since both seroconversion and the presence of PRRSV-1 RNA in reproductive tissues were correlated with the course of infection but not with shedding in semen, local replication of the virus may take place in the reproductive tissue. Other studies also found that shedding in semen appeared to be of longer duration than that of viraemia (Christopher-Hennings et al., 1995a,^2001^). It is hypothesised that this is a consequence of an initially viraemic seed of PRRSV into the reproductive tissue and subsequent local replication of the virus (Prieto et al., 2003). Shedding in semen has also been suggested to be constant or intermittent according to strain variation and exposure dose of the virus (Han et al., 2013). Likewise, host factor variations such as breed and age may have an impact on the shedding (Sirinarumitr et al., 1998; Christopher-Hennings et al., 2001). Boars in this study were considerably older (15.4 months) than boars in the experimental studies (5–8 months) (Sur et al., 1997; Christopher-Hennings et al., 2001; Prieto et al., 2003; Han et al., 2013). Furthermore, some of the boars were Yorkshire and Landrace and thereby genetically different from the majority of the Durocs used in the experimental studies. Due to the individual variation in the shedding of semen among boars, it is difficult to investigate this topic. Several studies have taken the approach of taking the boars out during the course of infection to study the distribution of PRRSV at different time points and thereby describe the impact of time on the PRRSV distribution, resulting in small sample sizes for each observation (Sur et al., 1997; Christopher-Hennings et al., 2001; Shin and Molitor, 2002; Prieto et al., 2003). Other studies prioritised the sample size, thereby limiting the study to certain time points of the infection (Christopher-Hennings et al., 1998; Han et al., 2013). This study examined most boars at one time, which unfortunately might have resulted in an even higher interindividual variability of time of infection due to the different exposure periods of the included animals. Notably, at delivery time for semen, the majority of the boars used in this study might have been at a later stage in the course of infection [potentially ∼29 days for the early infected group (G1) and less than 18 days for the late infected group (G2)] and that semen was collected the day before the rest of the samples. In addition, individual studies have used different detection methods, such as virus isolation (Christopher-Hennings et al., 1998, 2001; Prieto et al., 2003; Han et al., 2013), conventional RT-nested PCR (Christopher-Hennings et al., 1995a,^1998^, ^2001^; Sur et al., 1997; Shin and Molitor, 2002), RT-PCR (as in our study), and immunohistochemical methods (IHC, *in situ* hybridisation) (Christopher-Hennings et al., 1998; Shin and Molitor, 2002; Han et al., 2013), which complicates the comparison of the results. Of these methods, virus isolation may be the best choice for detecting infectious viruses, although it is considered to be less sensitive than PCR methods (Christopher-Hennings et al., 2001). For example, RT-PCR showed good correlation with a swine bioassay and was superior to virus isolation (Christopher-Hennings et al., 1995b). The opposite is the case with the highly sensitive PCR techniques that may detect non-infectious PRRSV RNA, although a sample tested positive by PCR should be regarded as *potentially* infectious. In this study, RT-PCR PRRSV RNA positive semen samples had relatively high Ct values (32.69–39.92, *M*: 35.67), and we did not succeed in propagating the virus in cell culture.

One of the boars in the early infected group did not seroconvert after virus exposure, which has previously been described in vaccine trials (Díaz et al., 2020; Pedersen et al., 2021). However, this result could also be false negative due to the relative high dilution of the serum in the ELISA or the boar could have had antibodies other than N protein detectable antibodies, e.g., neutralising antibodies. Furthermore, it is not known whether the four non-seroconverted boars in the late infected group, due to the time frame for seroconversion, were not infected early enough before slaughter to develop antibodies toward PRRSV. Most boars in experiential studies seroconvert between days 7 and 14 dpi, although it has been recorded as late as 21 dpi (Sur et al., 1997; Christopher-Hennings et al., 1998, 2001; Prieto et al., 2003; Han et al., 2013).

Porcine reproductive and respiratory syndrome virus 1 RNA was detected with approximately the same fraction in the caput, corpus, and cauda epididymis. Other studies have found a higher fraction of PRRSV-1 in the caput, followed by the corpus and cauda epididymis from day 2 until day 23 dpi, which was explained by the diverse blood supply to the cephalic region of the epididymis compared to the corporal and caudal regions (Prieto et al., 2003). The finding seems to be inconsistent since two other studies were not able to isolate the virus from the reproductive associated glands and tissue. Similarly, RT-nested PCR revealed positive results, indicating the presence of PRRSV RNA in these tissues (Christopher-Hennings et al., 1998, 2001). The latter study tested tissue from boars at 40–88 dpi, which could be a reason for the failure to detect replicable viruses.

Contamination of semen with PRRSV-1 could take place across several different epithelial surfaces from Sertoli cells in the testis, through the epididymis or on its route through the urethra. Only a small fraction (2–5%) of the semen comes from the ejaculate of the testis. The rest of the secretion originates from the prostate (55–75%), seminal vesicles (15–20%), and the Cowper’s gland (10–25%) (Dyce et al., 2002). Therefore, there is a theoretical possibility that semen is contaminated with PRRSV by other secretory glands than the testis. Different results are presented in the literature with regard to the origin of infectious locations in the reproductive tissue. Han et al. (2013) failed to isolate any PRRSV in adjacent glands (prostate, bulbourethral, and vesicular) but still detected PRRSV-specific *in situ* hybridisation signals in both the ductus deferens and prostate gland (Han et al., 2013). Prieto et al. (2003) isolated PRRSV sporadically from prostate glands (25%), bulbourethral glands (7,5%) and vesicular glands (5%), which was more often than in the testis (5%). Christopher-Hennings et al. (2001) constantly detected, with few exceptions, PRRSV RNA in the adjacent reproductive tissue at 21 dpi, but only sporadic at 40–47 dpi, and not at 61 or 88 dpi.

A complex interaction between immunomodulatory molecules, a physical barrier (tight junctions and basolateral and apical membranes) between adjacent Sertoli cells (testis) or epithelial cells (epididymis) and physiological properties constitute the dynamic blood-testis-barrier (BTB) and blood-epididymis-barrier (BEB), which allows a microenvironment with foreign post-meiotic spermatids to be present in the adluminal space (Francavilla et al., 1999; Fijak et al., 2011; Meinhardt and Hedger, 2011; Kaur et al., 2013, 2014; Mao et al., 2018). However, the BTB and BEB might, to some extent, be immunologically incomplete (Yule et al., 1988; Cheng and Mruk, 2012). Therefore, the interstitial space is still a local immunosuppressive milieu caused by androgens (testosterone) (Cutolo et al., 2004) and anti-inflammatory cytokines (Avallet et al., 1994; Mital et al., 2011). Macrophages are resident in the testicular interstitium (Hedger, 2002), and in rodent models, for instance, they contribute to this immunosuppression with the secretion of immunosuppressive cytokines (Kern et al., 1995; Bryniarski et al., 2004; Winnall et al., 2011). Paradoxically, since PRRSV has a trophism for the macrophage lineages (Halbur et al., 1995; Rossow et al., 1995; Mengeling et al., 1996; Cheon et al., 1997; Sur et al., 1998; Han et al., 2013), these could contribute to the maintenance of PRRSV in the male reproductive tissue (Shin and Molitor, 2002), although macrophages/mononuclear cells should not be present in the adluminal compartment of the testicular or epididymal tubules.

Reinforcing the hypothesis that macrophage/mononuclear cells contribute to the contamination of semen with PRRSV-1 is relevant in this study, since mononuclear cells and MGC were present in the adluminal compartment of the testis and epididymis in PRRSV-infected boars and not in the control boars (*p* < 0.05). This is associated with a high degree of uncertainty, since this finding was only observed in a small proportion of the boars, and it could be influenced by circumstances pertaining to a natural infection, as mentioned earlier (unknown infectious virus dose, time of infection, and time frame of exposure). Potentially infected cells in the adluminal compartment of the testis and/or epididymis were seen in both infected boar groups (G1 and G2), and MGC has also been found in seminiferous tubules before (Sur et al., 1997). Unfortunately, none of these cells in the adluminal compartment revealed positive IHC signals in this study, thereby confirming the presence of PRRSV-1 in the unique semen-supplying environment. Therefore, the final connection between naturally PRRS-infected boars and venereal transmission remains unresolved. IHC-positive cells were detected in the interstitium of the testis and epididymis in the early infected group, at which time contamination of semen may have occurred. In the detection of PRRSV-1-infected cells by IHC, the representative histological sections were 4–5 μm, and, compared with the infrequent and sporadic detection of PRRSV in semen by RT-PCR with high Ct values, the possibility of finding IHC-marked cells in the adluminal compartment is small. In our study, all IHC-positive cells were macrophages, whereas in other studies, also spermatogonia, spermatocytes, and spermatids have also been found sporadically positive (Sur et al., 1997; Christopher-Hennings et al., 1998; Shin and Molitor, 2002; Han et al., 2013). PRRSV has also been found in the semen of vasectomised boars, which rules out sperm cells as the only source of contamination. Therefore, due to the pathogenesis associated with the hematogenous spread of infected macrophages, viraemia and shedding in semen seem to be correlated in early infected boars. Since this has not been addressed in several of the earlier studies, it is tempting to speculate that interstitial macrophages could be the source of prolonged shedding in semen in the case of passed viraemia, as indicated by the IHC results in this study.

This study contributes to establishing the correlation between findings in experimentally and naturally PRRSV-infected boars, since the findings of potentially PRRSV-1-infected foreign cells within the adluminal compartment of the testis and epididymis were determined. IHC-positive cells in the adluminal compartment were not determined; only findings of PRRSV-infected macrophages in the interstitium of the testis and epididymis were detected. The presence of PRRSV-1 RNA in reproductive tissue is more evident in the acute phase of infection, even though there does not seem to be a link between seroconversion, PRRSV-1 RNA in tissue, and shedding in semen, which also occurs in the chronically PRRSV-infected and seroconverted boars. This needs to be addressed in surveillance programmes.

## Data Availability Statement

The raw data supporting the conclusions of this article can be shared by the authors on request, without undue reservation.

## Ethics Statement

Ethical review and approval was not required for the animal study because all samples were obtained during disease investigation or from commercially slaughtered animals. Written informed consent was obtained from the owners for the participation of their animals in this study.

## Author Contributions

CK and LL conceived of the study and developed the approach. KP and LS along with veterinarians, technicians, and farm personnel, collected the samples. LK carried out all microbial laboratory work. SB-P did the histological preparation and examination under supervision of HJ. KP processed the data. LS under supervision of CK and LL. KP wrote the draft manuscript. All authors contributed to the drafting, revision of the manuscript, read and approved the final manuscript.

## Conflict of Interest

KP, CK, and LS worked for SEGES Danish Pig Research Centre at the time of sample collection and during the investigation. The remaining authors declare that the research was conducted in the absence of any commercial or financial relationships that could be construed as a potential conflict of interest.

## Publisher’s Note

All claims expressed in this article are solely those of the authors and do not necessarily represent those of their affiliated organizations, or those of the publisher, the editors and the reviewers. Any product that may be evaluated in this article, or claim that may be made by its manufacturer, is not guaranteed or endorsed by the publisher.
