# Supplementary material for: Virological and Histopathological Findings in Boars Naturally Infected With Porcine Reproductive and Respiratory Syndrome Virus Type 1
Source: Front Microbiol. 2022 May 11;13:874498. doi: 10.3389/fmicb.2022.874498 (PMC9130840; doi:10.3389/fmicb.2022.874498)
Supplement: Supplementary file 1 [file Data_Sheet_1.PDF]

Supplementary Material

Table S1 – Overview of binary results (+/-) in the PRRSV RT-qPCR in all sample material (serum, semen, testis and caput, corpus and cauda of epididymis) from PRRSV-infected boars. Results are given in “All” (total) and groupwise (G1, early infected boars and G2, later infected boars). Results are given in quantity and percentage.

	PRRSV RNA	*All*	*Serum*	*Semen*	*Testis*	*Epididymis*			
						All	*Caput*	*Corpus*	*Cauda*
All	+	88 (43%)	18 (51%)	7 (22%)	16 (46%)	47 (45%)	16 (46%)	15 (43%)	16 (46%)
	-	119 (57%)	17 (49%)	25 (78%)	19 (54%)	58 (55%)	19 (54%)	20 (57%)	19 (54%)
G1	+	28 (25%)	5 (26%)	3 (17%)	5 (26%)	15 (26%)	4 (21%)	5 (26%)	6 (32%)
	-	85 (75%)	14 (74%)	15 (83%)	14 (74%)	42 (74%)	15 (79%)	14 (74%)	13 (68%)
G2	+	60 (64%)	13 (81%)	4 (29%)	11 (69%)	32 (67%)	12 (75%)	10 (63%)	10 (63%)
	-	34 (36%)	3 (19%)	10 (71%)	5 (31%)	16 (33%)	4 (25%)	6 (37%)	6 (38%)
Total	*+ and -*	207 (100%)	35 (100%)	32 (100%)	35 (100%)	105 (100%)	35 (100%)	35 (100%	35 (100%)

Figure S1 – Study timeline with calendar day of sample collection and study day (Dx)

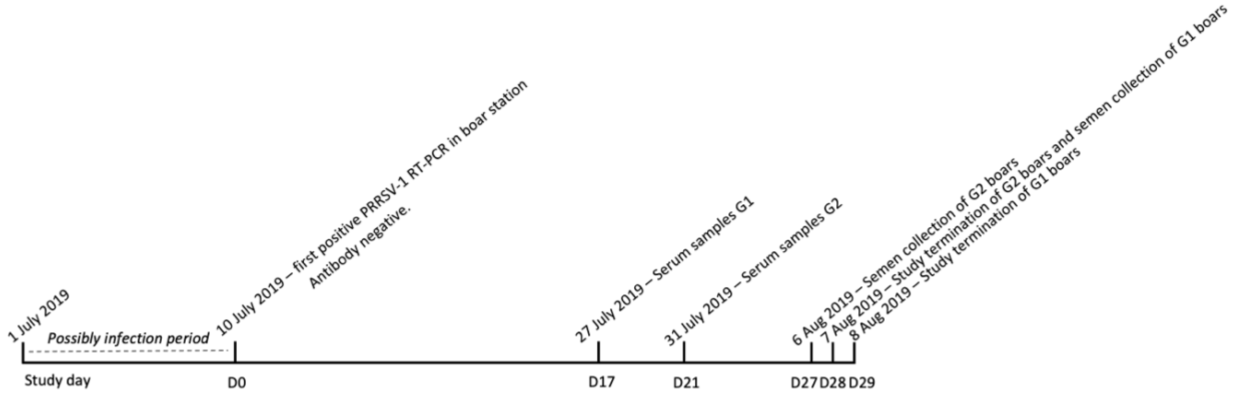

Figure S2 – Diagram of the housing unit with PRRSV-infected boars. "Stald" indicates section, "gang" indicates corridor and "lab" indicates laboratories.

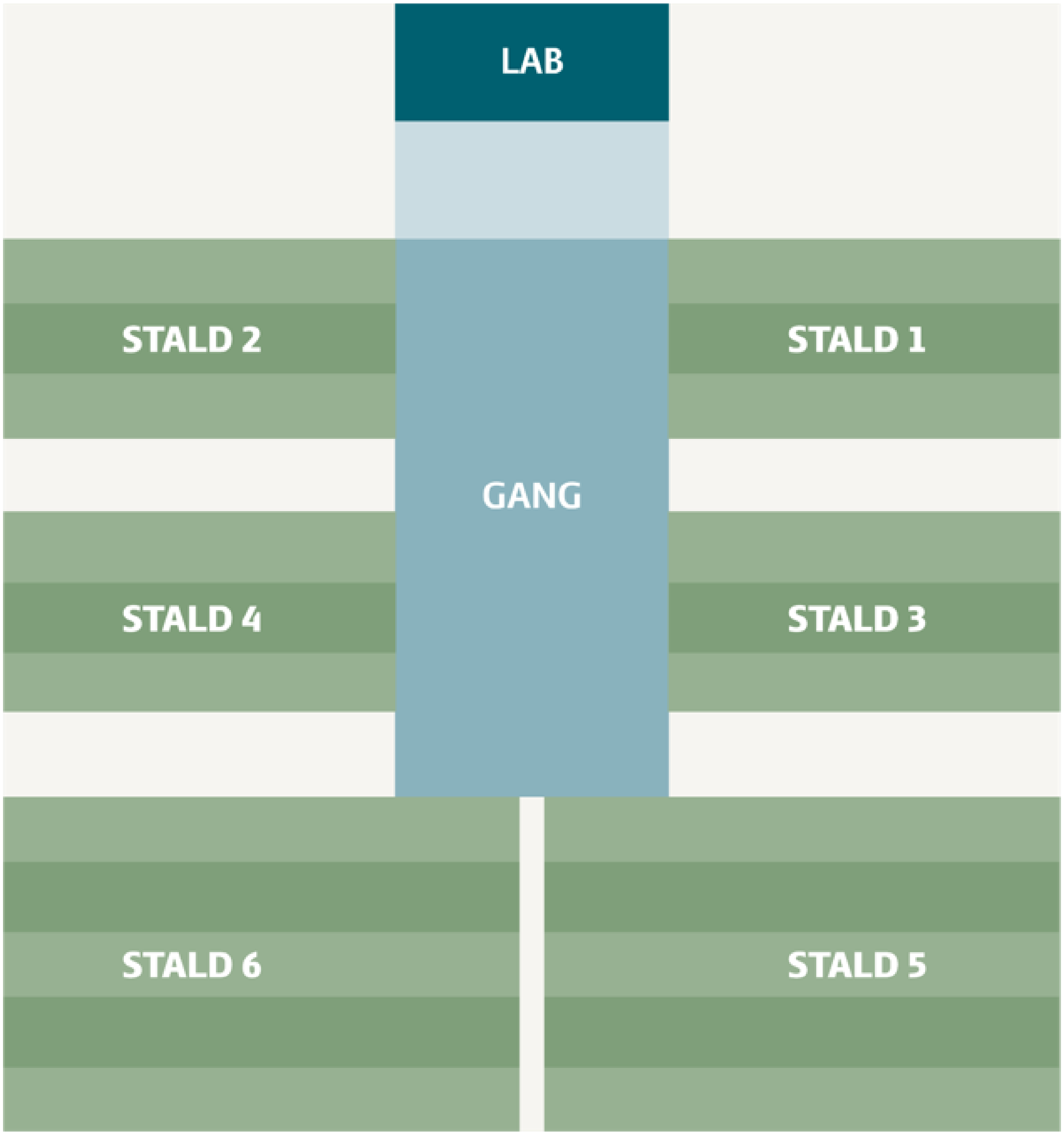

Figure S3 - Histological sections of tissue from control boars. A: Interstitial oedema (OE) in the epididymis (x10 objective). B: Severe vacuolization of the epididymal epithelium (x20 objective). C: Focal tubular necrosis (arrows) of testis and hyperaemia (x10 objective). DS: degenerated/detached spermatocytes and epithelial lining cells. Haematoxylin and eosin stain.

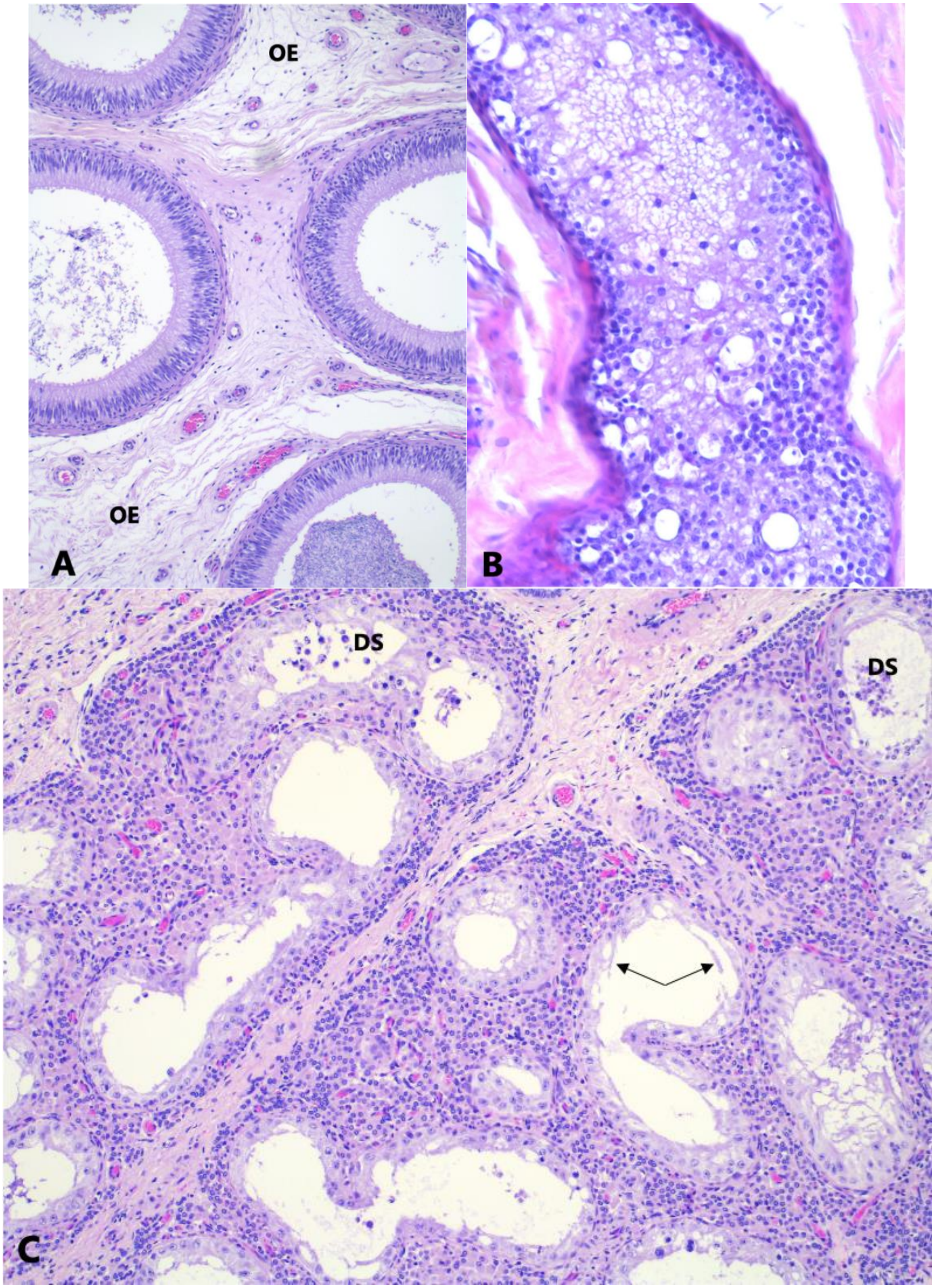

Figure S4 - Histological sections of tissue from PRRSV-1-infected boars. a: Oedema in testis. b: Oedema in epididymis. c: Dilated lymph vessel in testis. d: Pyknosis and karyorrhexis of the spermatocytes in testis. e: Leydig cells with absent nuclei in testis. f: Hyperaemia of testis.

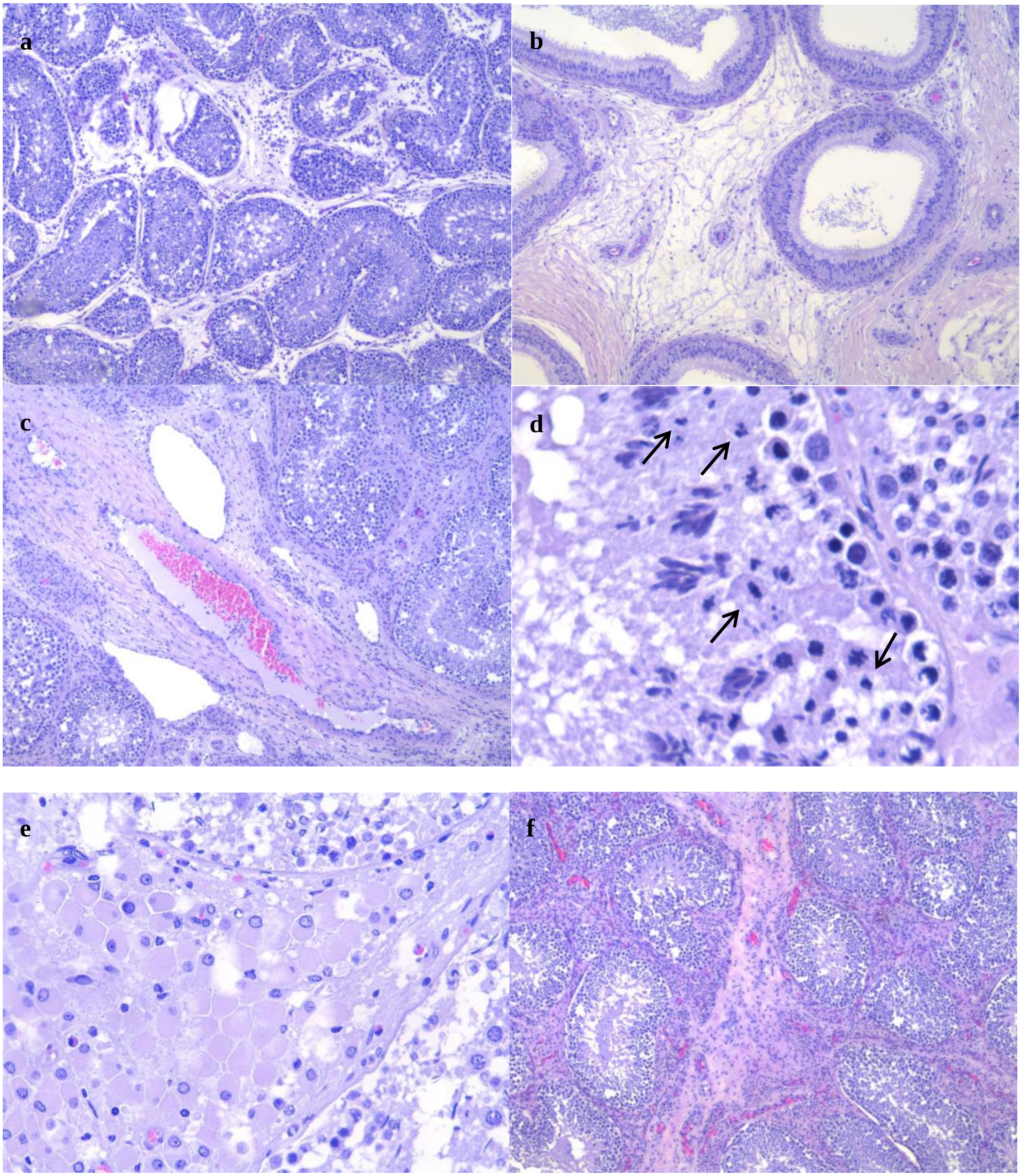

Figure S5 - Histological sections of tissue from PRRSV-1-infected boars. a + b: Mononuclear cell infiltration of testis. c + d: Interstitial mononuclear cell infiltration of epididymis.

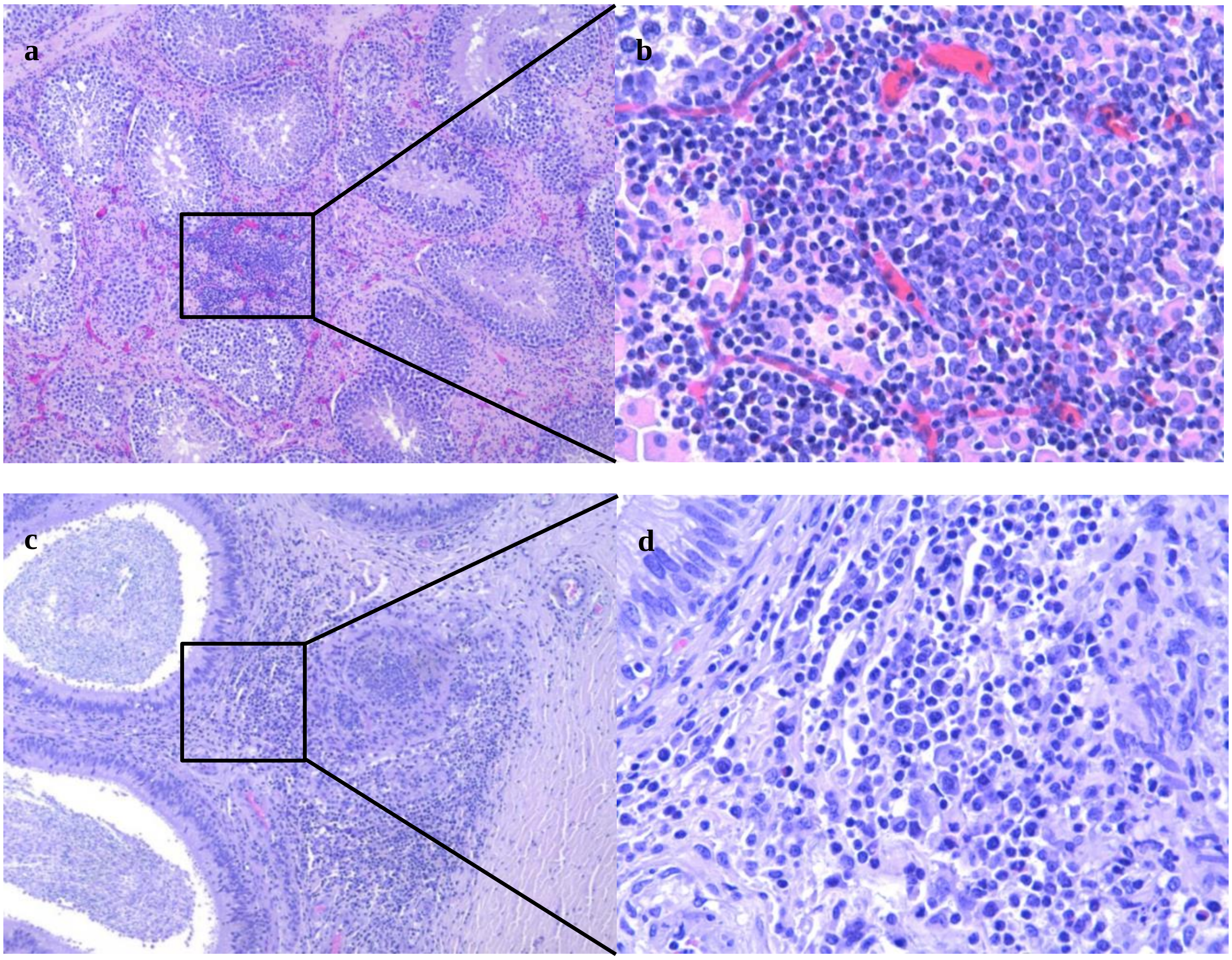

Figure S6 - Histological sections of tissue from PRRSV-1-infected boars. Focal tubular necrosis/degeneration of testis (a + b). Smooth muscle hypertrophy in epididymis (c, HE staining + d, Masson Trichrome staining).

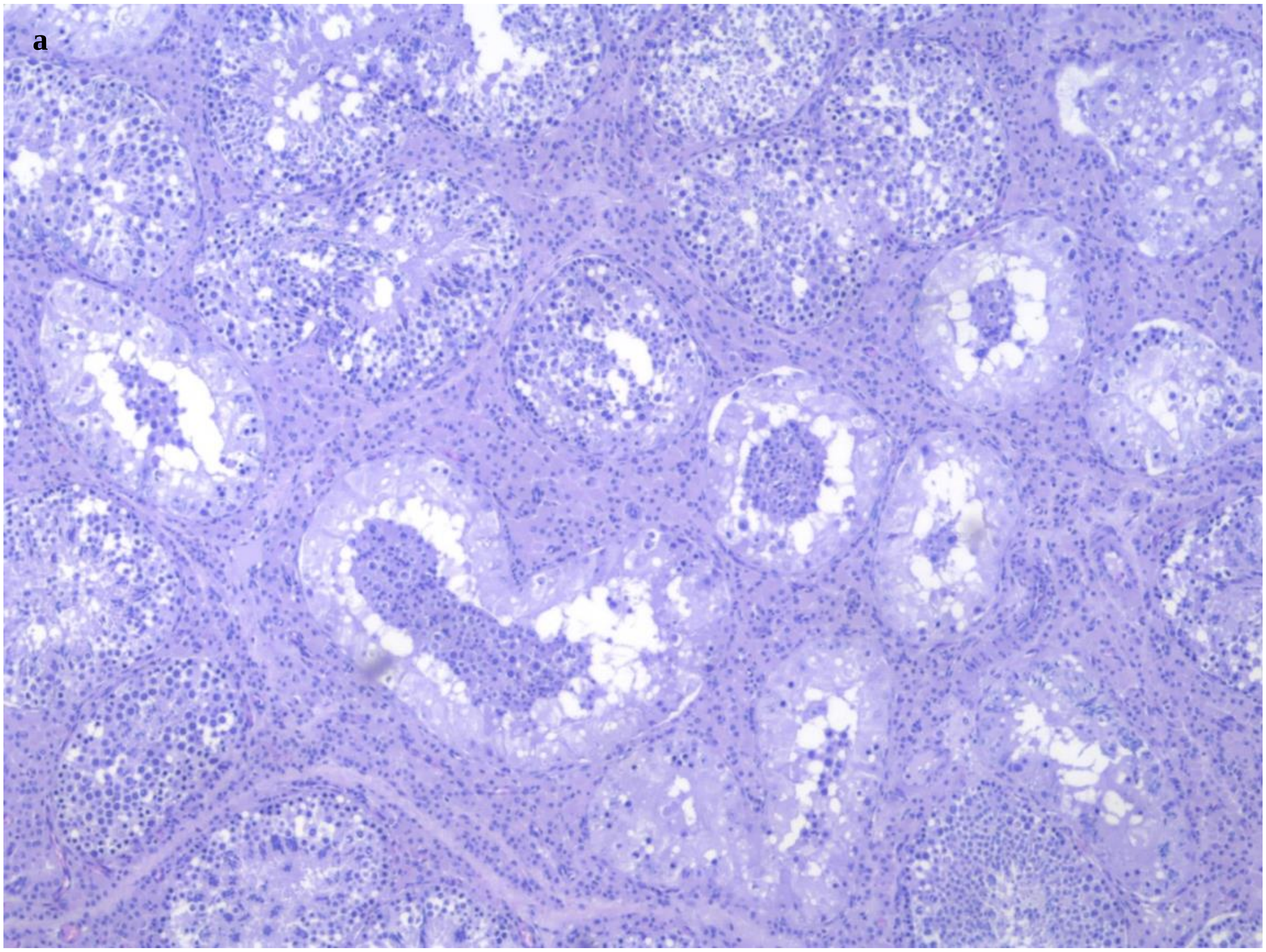

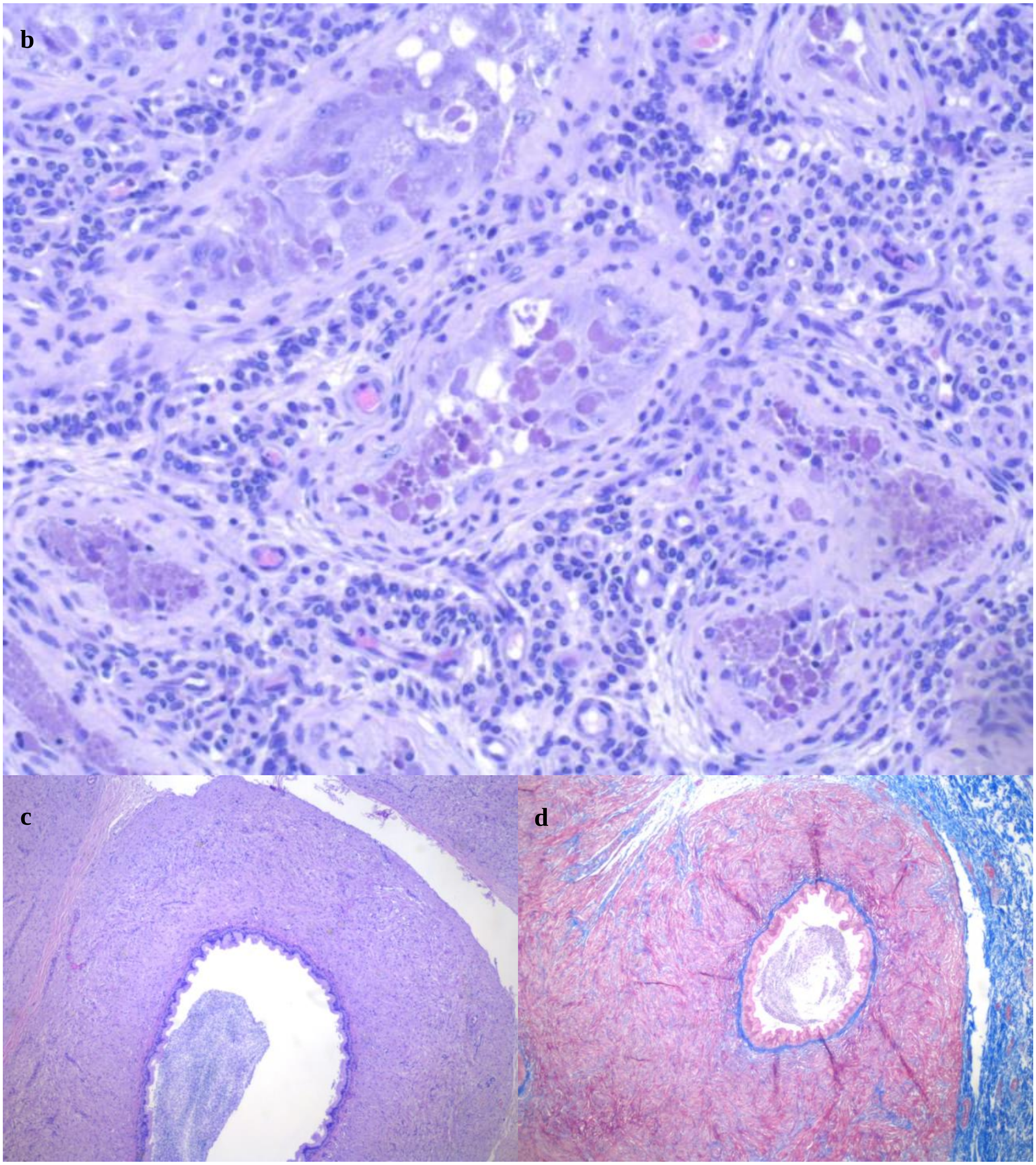
b
c
d

Figure S7 - Histological sections of tissue from PRRSV-1-infected boars. a: Severe vacuolisation of the epididymal epithelium. b: Intraluminal swollen/enlarged/giant cells in epididymis.

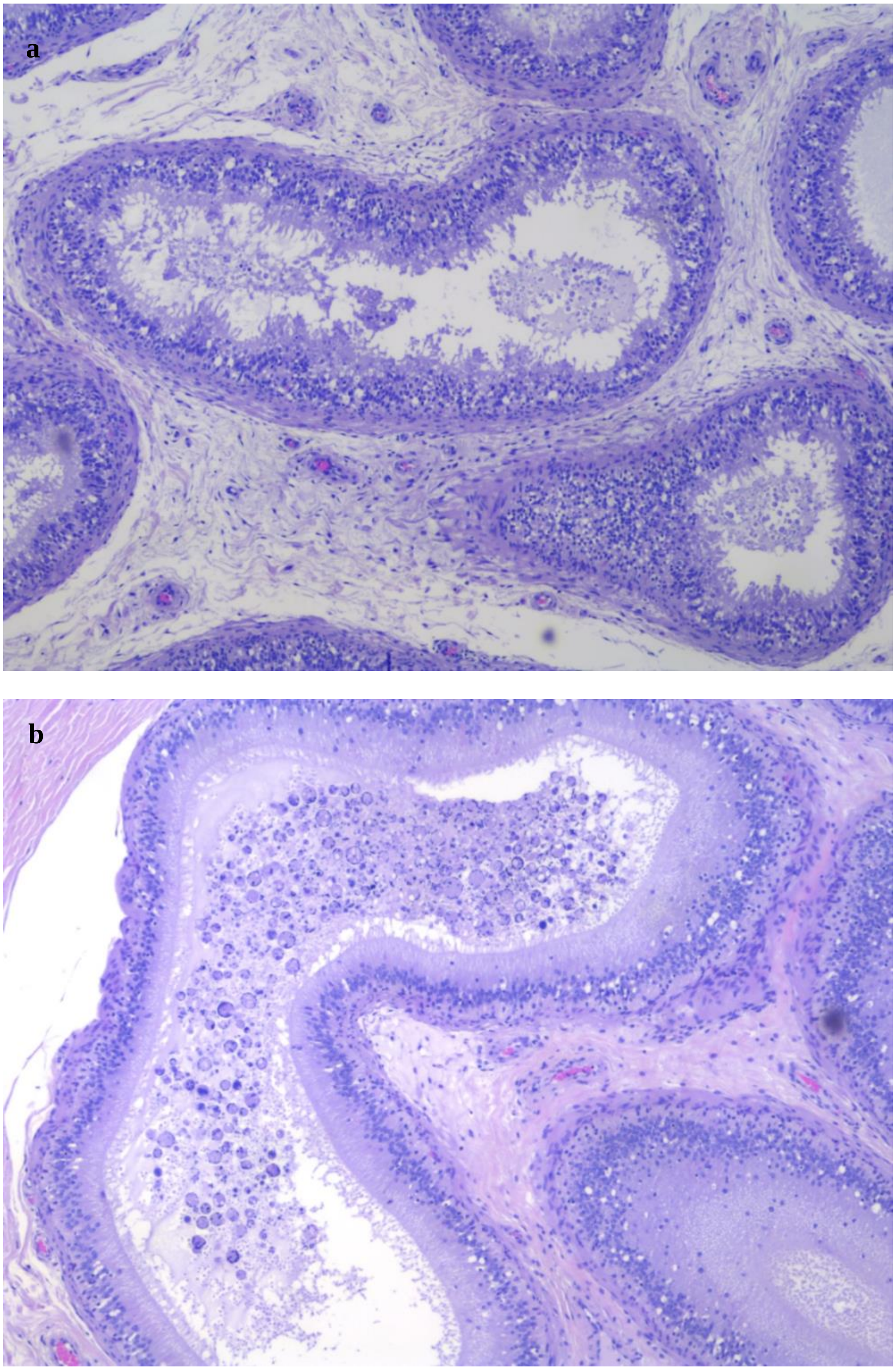

Figure S8 - Histological sections of IHC stained tissue from PRRSV-1-infected boars. a: (40X) Few, focally distributed, infected macrophages in the interstitium of epididymis and b: (20X) Few, focally distributed, infected macrophages in the interstitium of epididymis. c: (40X) Few, focally distributed, infected macrophages in the interstitium of epididymis.

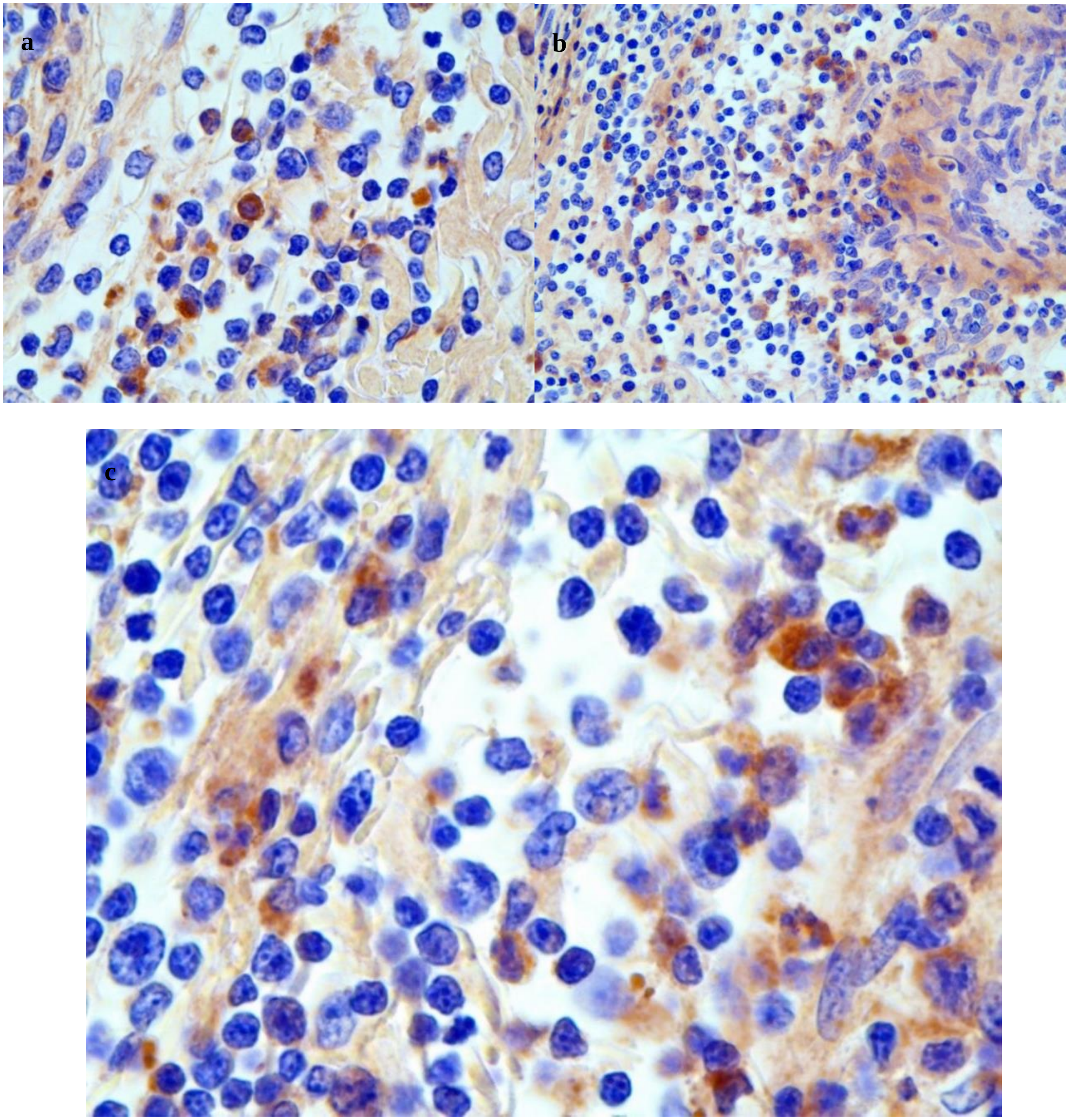

Figure S9 - Histological sections of IHC positive and negative control lung tissue. A: IHC-positive lung tissue from a PRRSV-1 positive boar as positive control. Immunohistochemistry stain towards PRRSV-1. B: Negative control tissue. Nonsense stain (X0903). x40 objective.

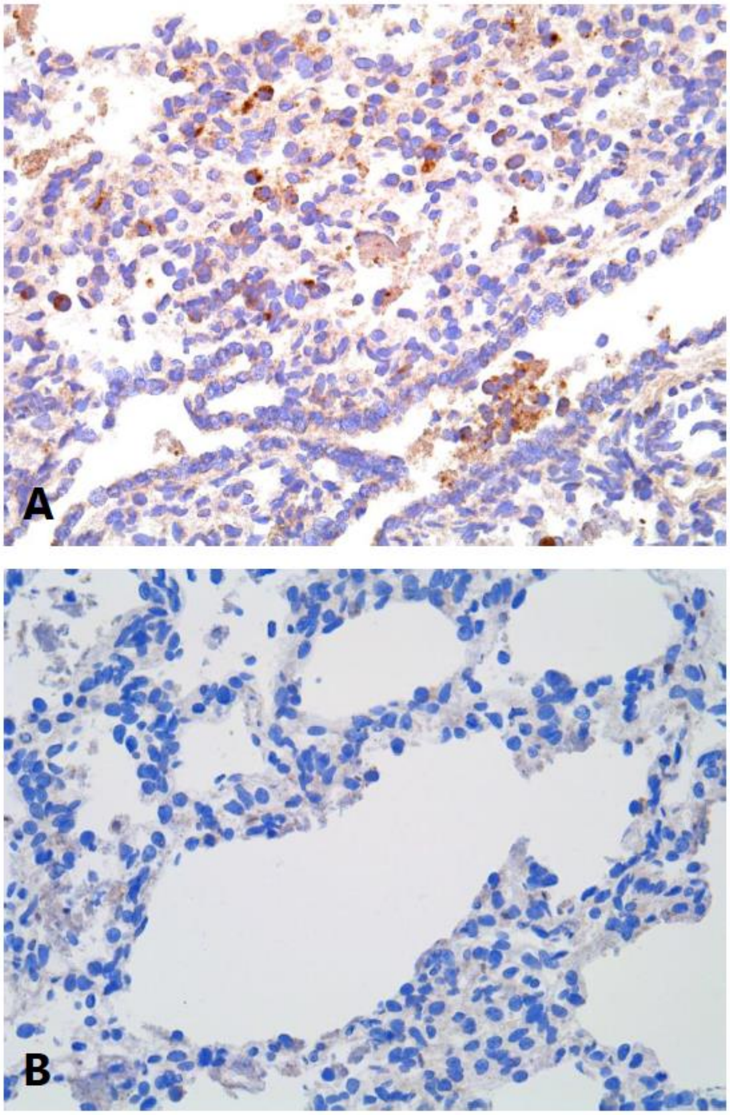

Table S2 – Real time RT-PCR results (Ct values) of the RNA cell isolates from the propagation of virus in each passage (P).

Tissue:	P1	P2	P3	Semen	P1	P2	P3
1620	0.00	0.00	0.00		-	-	-
8543	0.00	0.00	0.00	8543	0.00	0.00	0.00
5300	0.00	0.00	0.00	5300	0.00	0.00	0.00
6560	0.00	0.00	0.00	6560	0.00	0.00	0.00
8540	34.99	0.00	0.00	8540	0.00	0.00	0.00
7794	0.00	0.00	0.00	7794	34.95	41.23	0.00
